# Visual Detection of Human Antibodies Using Sugar Chain-Immobilized Fluorescent Nanoparticles: Application as a Point of Care Diagnostic Tool for Guillain-Barré Syndrome

**DOI:** 10.1371/journal.pone.0137966

**Published:** 2015-09-17

**Authors:** Hiroyuki Shinchi, Nobuhiro Yuki, Hideharu Ishida, Koichi Hirata, Masahiro Wakao, Yasuo Suda

**Affiliations:** 1 Department of Chemistry, Biotechnology and Chemical Engineering, Kagoshima University, 1-21-40 Kohrimoto, Kagoshima 890–0065, Japan; 2 Department of Neurology, Dokkyo Medical University, Tochigi 321–0293, Japan; 3 Brain & Mind Research Institute, University of Sydney, Level 7, Building F, 94 Mallett Street, Camperdown, NSW 2050, Australia; 4 Department of Applied Bioorganic Chemistry, Gifu University, Gifu 501–1193, Japan; 5 SUDx-Biotec Corporation, 1-42-1 Shiroyama, Kagoshima 890–0013, Japan; Medical University of Innsbruck, AUSTRIA

## Abstract

Sugar chain binding antibodies have gained substantial attention as biomarkers due to their crucial roles in various disorders. In this study, we developed simple and quick detection method of anti-sugar chain antibodies in sera using our previously developed sugar chain-immobilized fluorescent nanoparticles (SFNPs) for the point-of-care diagnostics. Sugar chain structure on SFNPs was modified with the sugar moieties of the GM1 ganglioside via our original linker molecule to detect anti-GM1 antibodies. The structures and densities of the sugar moieties immobilized on the nanoparticles were evaluated in detail using lectins and sera containing anti-GM1 antibodies from patients with Guillain-Barré syndrome, a neurological disorder, as an example of disease involving anti-sugar chain antibodies. When optimized SFNPs were added to sera from patients with Guillain-Barré syndrome, fluorescent aggregates were able to visually detect under UV light in three hours. The sensitivity of the detection method was equivalent to that of the current ELISA method used for the diagnosis of Guillain-Barré syndrome. These results suggest that our method using SFNPs is suitable for the point-of-care diagnostics of diseases involving anti-sugar chain antibodies.

## Introduction

Sugar chains found on cell surfaces are involved in various biological processes, such as cell signaling, cell-cell recognition, cancer and immunity. Because the structures and expression levels of sugar chains vary depending on the cell states and cellular environments, some sugar chains can be used as biomarkers [1]. Cancer cells produce various unique sugar chain markers including fucose-containing sugar chains in hepatic cancer [2–4]. Specific sugar chains also act as antigens that bind to natural or acquired antibodies to induce an immuno-compromised response [5–9]. The production of auto-antibodies against sugar chains occasionally leads to the development of severe autoimmune diseases, such as Guillain-Barré syndrome (GBS) [10, 11].

GBS is the most frequent cause of acute flaccid paralysis. A common misconception is that GBS has a good prognosis; however, up to 20% of patients remain severely disabled, and approximately 5% die [12]. One-third of patients with GBS develop the disease after infection by *Campylobacter jejuni*, which expresses a lipo-oligosaccharide that mimics sugar moieties of ganglioside [13, 14]. The lipo-oligosaccharide induces an immune response and the production of autoantibodies against sugar moieties of gangliosides, producing the autoimmune disease. Currently, an ELISA that uses GM1 and GD1a gangliosides to detect anti-ganglioside autoantibodies in sera is utilized to confirm the diagnosis of GBS because it is often misdiagnosed as stroke [15, 16]. Although early diagnosis and medication is important for GBS treatment, the ELISA for GBS is time consuming, and it can take several days to receive the assay results from a diagnostic laboratory. Therefore, a rapid, simple and point-of-care diagnostic test for GBS is required.

For detection of anti-sugar chain antibodies, several detection methods including carbohydrate microarray and ELISA have been utilized [17–19]. However, those methods are not suitable for diagnosis in clinical facilities (point-of-care diagnostics) because it needs mature techniques and/or special instruments. Recently, we developed sugar chain-immobilized fluorescent nanoparticles (SFNPs) as a simple interaction analysis tool for sugar chain binding proteins [20]. SFNPs can clearly visualize the interactions between sugar chains and sugar chain binding proteins by forming specific fluorescent aggregates under UV irradiation. Additionally, this method can be utilized for various types of sugar chain binding proteins because the sugar chain structure on the SFNPs can be easily modified. In this study, we applied our SFNP technology to a novel detection method for anti-sugar chain antibodies, in which anti-ganglioside antibodies in sera with GBS were visually detected in three hours as they form fluorescent aggregates with the SFNPs. This method is simple and quick compared with the ELISA method, and would be applicable for the point-of-care diagnostics.

## Materials and Methods

### Preparation of CdTe/CdS core/shell quamtum dots (QDs)

CdTe/CdS core/shell QDs stabilized with 3-MPA were prepared according to the method from our previous report, with a slight modification [20]. Te powder (16.0 mg, 0.125 mmol, Nakalai Tesque, Kyoto, Japan) and NaBH_4_ (18.9 mg, 0.50 mmol, Nakalai Tesque) were dissolved in argon-bubbled water (2 mL). The resulting mixture was stirred at room temperature for 1.5 h. In another flask, CdCl_2_ (9.17 mg, 50.0 μmol, Nakalai Tesque) and 3-MPA (5.45 μL, 63.0 μmol, Nakalai Tesque) were dissolved in water (10 mL), and the pH of the solution was adjusted to 9 using 1 M NaOH. Argon gas was then bubbled through the reaction mixture for 30 min with stirring. The resulting mixture was heated to 105°C and stirred vigorously. The NaHTe solution (200 μL) was quickly added to this mixture, and the resulting mixture was further stirred at the same temperature under open-air conditions. After stirred for 2 h, the CdTe solution was then cooled to room temperature. The CdTe QDs were then precipitated by adding 2-propanol, and the QDs were dissolved in water. The solution was left for 10 h at 4°C in the dark. A solution of thioacetamide (0.27 μL, 1.33 M) was then added to the CdTe solution. The reaction mixture was stirred at 105°C for 57 h and then cooled to room temperature to obtain the solution of CdTe/CdS core/shell QDs.

### Synthesis of sugar chain-ligand conjugate

GM1-Glc-f-mono was synthesized according to the methods described in our previous report [21]. The GM1-Glc sugar chain [22] (1.0 mg, 0.86 μmol) and f-mono [21] (0.28 mg, 1.1 μmol) were dissolved in water (20 μL) and *N*,*N*-dimethylacetamide (30 μL), respectively. Then, the GM1-Glc solution and acetic acid (6 μL) were added to the f-mono solution. After the reaction mixture was incubated at 40°C for 6 h, sodium cyanoborohydride (1.5 mg, 24 μmol, Sigma-Aldrich, St. Louis, MO, USA) in water (10 μL) was added. The reaction mixture was left at 40°C for 3 days and then lyophilized. The residue was then dissolved in water and purified over an ODS column (eluted with 6:4 water:methanol) to obtain the desired GM1-Glc-f-mono as a white powder. Yield: 0.69 mg (56%). MALDI-TOF MS calculated for C_56_H_92_N_5_O_34_S_2_: 1442.51; found: *m/z* 1442.48 [M-H]^−^.

### Immobilization of sugar chains onto the CdTe/CdS QDs

Sugar chains were immobilized onto the CdTe/CdS QDs according to the methods described in our previous report, with a slight modification [20]. GM1-Glc-f-mono (1 mM, 50 μL), and an aqueous solution of NaBH_4_ (10 mM, 50 μL) were mixed at room temperature, and then the mixture was left for 10 min. Next, a CdTe/CdS QDs solution (1.8 μM, 100 μL) was added to the mixture, which was then stirred for 24 h at room temperature in the dark. Excess ligand conjugates were removed by centrifugal filtration (14,000 *g*, 5 min) using an Amicon Ultra-10K (Millipore, MA, USA). The residue was washed with water three times, and then, PBS was added to prepare the GM1-SFNP solution.

### Analysis of binding interactions between proteins and GM1-Glc-SFNPs

Each protein (Con A, RCA120, PNA, WGA, and BSA) was dissolved in PBS, and each protein solution (5 μL, 3.6 μM) was placed in a microtube. Then, the SFNP solution (5 μL, 0.2 μM) was added to each microtube. After incubating for 1 h in the dark, the mixture was centrifuged at 14,000 *g* for 5 min. Then, the fluorescence spectrum (excitation wavelength at 360 nm) of the supernatant from each tube was measured.

### Use of GM1-Glc-SFNPs to detect anti-ganglioside antibodies in sera from patients with GBS

The sera used in this study were supplied from patients with GBS. Before being treated, all patients or their family in some cases agreed to the written informed consent from the Dokkyo University Hospital that samples from patients may be used for the clinical or preclinical study performed by the Department of Neurology, Dokkyo Medical University. The study was evaluated and approved by the Ethical Committee of Dokkyo Medical University (No. 1973). Serum from a GBS patient (5 μL) and a GM1-Glc-SFNPs solution (5 μL, 0.1 μM) were mixed in a microtube. After overnight incubation at 4°C in the dark, the mixture was centrifuged at 14,000 *g* for 5 min. Fluorescent aggregates were observed under UV irradiation, and the fluorescent spectrum of the supernatant from each sample was measured.

### SDS-PAGE and western blotting of aggregates of GM1-Glc-SFNPs and anti-GM1 antibodies

The aggregates of GM1-Glc-SFNPs and anti-GM1 antibodies in sera from patients with GBS were collected, washed three times with PBS, and then, dispersed in PBS. The dispersed solution was analyzed using SDS-PAGE and a 10% polyacrylamide gel stained with silver under reducing conditions or an 8% polyacrylamide gel under non-reducing conditions according to the typical procedure. Then, typical western blotting was performed to transfer proteins from the gel to a PVDF membrane. The membrane was then blocked with 5% skimmed milk in PBS with 0.05% Tween 20 (PBS-T) for 1 h. After washing with PBS-T three times, the membrane was incubated in a solution of HRP-conjugated anti-human IgG antibody (goat) and 5% skimmed milk in PBS-T (1:5000) for 1 h at room temperature. The gel was then developed using Chemi-Lumi One (Nakalai Tesque).

### Inhibition of the agglutination assay using GM1 sugar chains

Patient serum (2.5 μL), a GM1-Glc-SFNP solution (5 μL, 0.1 μM), and a GM1 sugar chain solution (2.5 μL, 1–16 mM) were mixed in a microtube. After incubating for 6 h at 4°C in the dark, the mixture was centrifuged at 14,000 *g* for 5 min. Aggregate formation was evaluated under UV irradiation, and the fluorescent spectrum of the supernatant of each tube was measured.

### SFNPs agglutination assay for 100 samples of sera from patients with suspected GBS

A GM1-Glc/TEG(5:5)-SFNPs or GM1-SFNPs solution (15 μL, 0.1 μM) and serum (15 μL) were mixed and incubated for 3 h at 4°C in the dark, and the mixture was centrifuged at 14,000 *g* for 5 min. The fluorescent aggregates were visually examined under UV irradiation. Titers of serum IgG antibodies to gangliosides were determined by ELISA. Each serum was diluted at 1:500, and titers were graded as described previously [23]: An optical density at 492 nm of less than 0.1 was judged to be negative. The optical density of 0.1 to 0.5 was categorized as 1+; 0.5 to 1.0, 2+; 1.0 to 1.5, 3+; 1.5 to 2.0, 4+; 2.0 to 2.5, 5+; and 2.5 or more, 6+ (S1 and S2 Tables).

### Statistics

Differences in proportions were analyzed by the Fisher’s exact test using 2×2 tables. The *p*-values less than 0.05 were considered statistically significant.

## Results

### Preparation and binding experiments with SFNPs containing the GM1 sugar moieties

**Fig 1:**
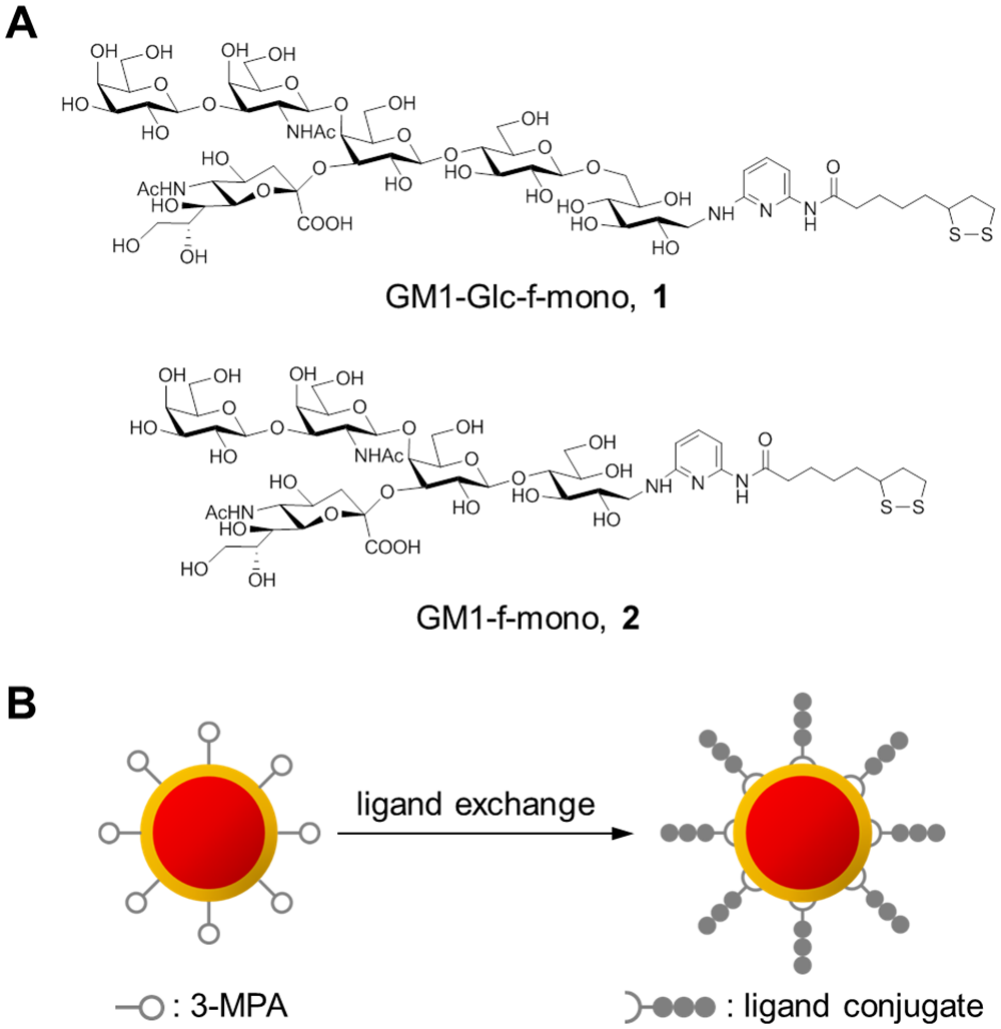
Preparation of GM1-Glc-immobilized fluorescent nanoparticles (GM1-Glc-SFNPs). (A) The structures of synthesized ligand conjugates containing GM1 sugar chain moieties. (B) Scheme showing the preparation of SFNPs containing GM1 sugar moieties.

**Fig 2:**
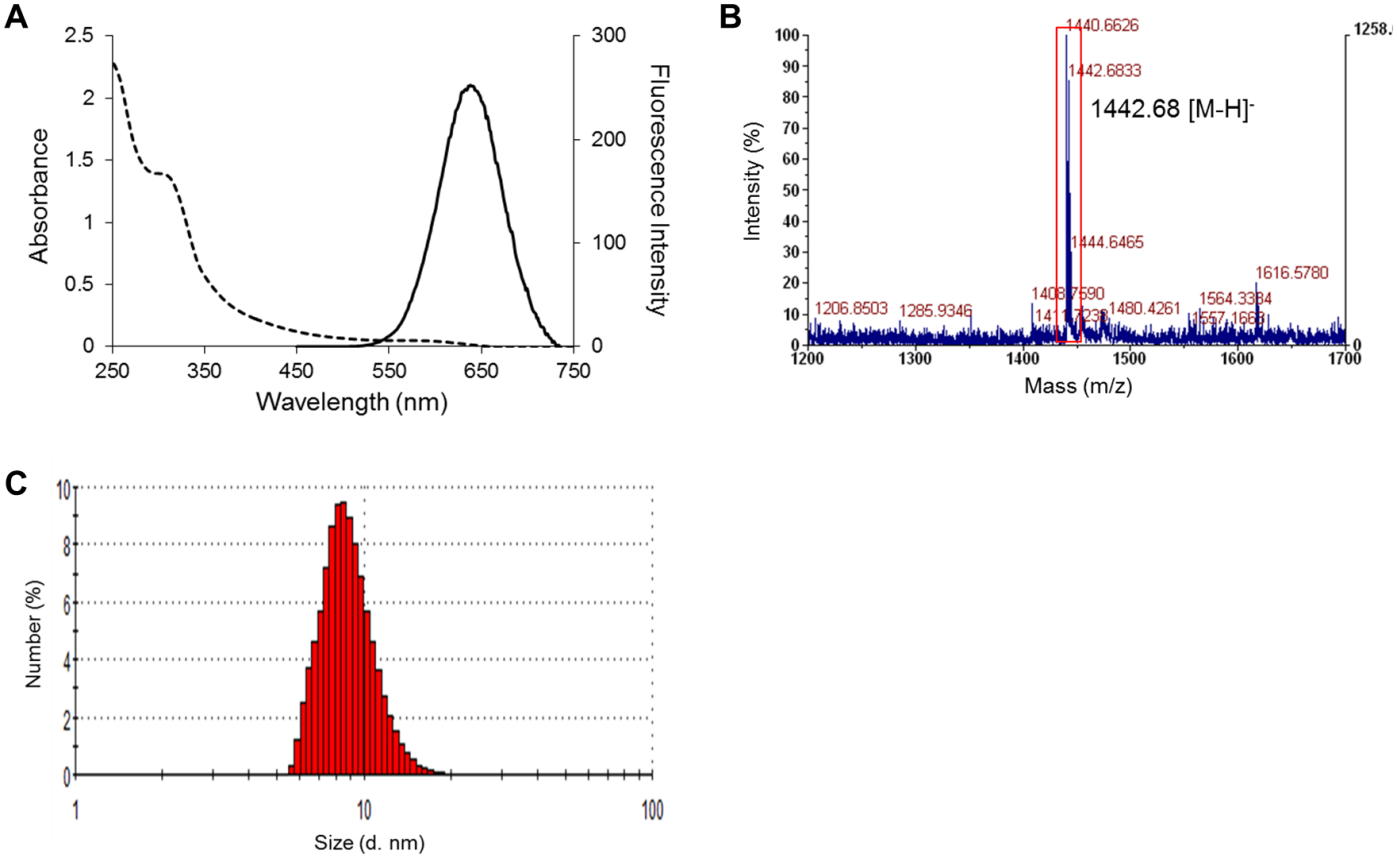
Characterization of GM1-Glc-immobilized fluorescent nanoparticles (GM1-Glc-SFNPs). (A) UV-Vis (dotted line) and fluorescence (solid line) spectra of GM1-Glc-SFNPs. (B) Dynamic light scattering measurement of GM1-Glc-SFNPs. The average hydrodynamic diameter was 8.9 nm. (C) MALDI-TOF MS analysis of GM1-Glc-SFNPs. The detected peak was *m/z*: 1442.68 [M-H]^−^, corresponding to GM1-Glc-f-mono.

To develop a diagnostic test for GBS, fluorescent nanoparticles containing GM1 sugar-chain moieties were designed. Sugar chain-ligand conjugates **1** and **2** were used as the GM1 sugar moieties. The conjugates were prepared by reductive amination of a chemically synthesized GM1-Glc sugar component [22] and a commercially available GM1 sugar component (ELICITYL, Crolles, France), respectively, with a fluorescent linker molecule (f-mono, Fig 1) [21]. The preparation of SFNPs with these conjugates was performed using our previous method, with a slight modification (Fig 1B). CdTe/CdS core/shell quantum dots (CdTe/CdS QDs) were used as fluorescent nanoparticles [20]. Ligand exchange reactions of CdTe/CdS QDs with GM1-Glc-f-mono **1** and GM1-f-mono **2** produced GM1-Glc-SFNPs and GM1-SFNPs, respectively. Both of the SFNPs showed distinct absorption patterns at approximately 310 nm, derived from the f-mono linker, and at approximately 600 nm, derived from the QD, as well as strong red fluorescence (Ex: 400 nm, Em: 650 nm; Fig 2A). The immobilization of GM1-Glc-f-mono on the SFNP was qualitatively confirmed using MALDI-TOF MS analysis (Fig 2B). The content of the GM1-Glc sugar chains was determined as 0.58 mg/mg of GM1-Glc-SFNP using the ABEE method [24]. The concentration of the SFNP solution was calculated from its absorption at 600 nm and was adjusted to approximately 0.1 μM with PBS [25]. The diameter of the GM1-Glc-SFNP was measured as 8.9 nm using dynamic light scattering (Fig 2C). GM1-SFNP was also validated as above (S1 Fig).

### Binding experiments of SFNPs containing GM1 sugar chains with lectins

**Fig 3:**
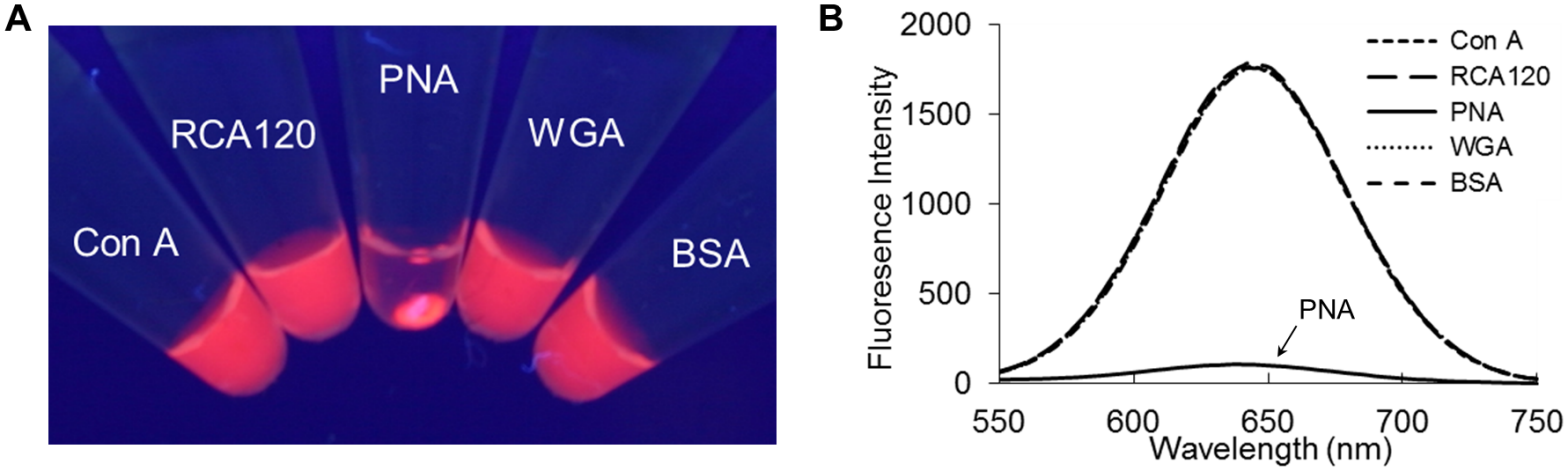
Interaction analysis of GM1-Glc-immobilized fluorescent nanoparticles (GM1-Glc-SFNPs) with lectins. (A) Analysis of interactions between GM1-Glc-SFNPs and lectins. Visual image of the mixture of GM1-Glc-SFNPs and proteins under UV irradiation. (B) Fluorescence spectra of the supernatant monitored by an excitation wavelength at 360 nm.

To investigate the functionality of the sugar chains on the prepared SFNPs, we examined the binding interactions of GM1-Glc-SFNPs with various types of lectins, a sugar chain-binding protein. Concanavalin A (Con A; known to specifically bind to αGlc- and αMan-), *Ricinus communis* agglutinin I (RCA120; known to specifically bind to βGal), peanut agglutinin (PNA; known to specifically bind to Galβ1-3GalNAc), wheat germ agglutinin (WGA; known to specifically bind to GlcNAc), and bovine serum albumin (BSA; known not to bind to sugar chains) were used. GM1-Glc-SFNP specifically interacted only with PNA. Fluorescent aggregates were produced in this process (Fig 3A), and the fluorescence intensity of the supernatant was specifically decreased (Fig 3B). Similar results were obtained with GM1-SFNPs (S1 Fig).

### Agglutination assay using sera from patients with GBS

**Fig 4:**
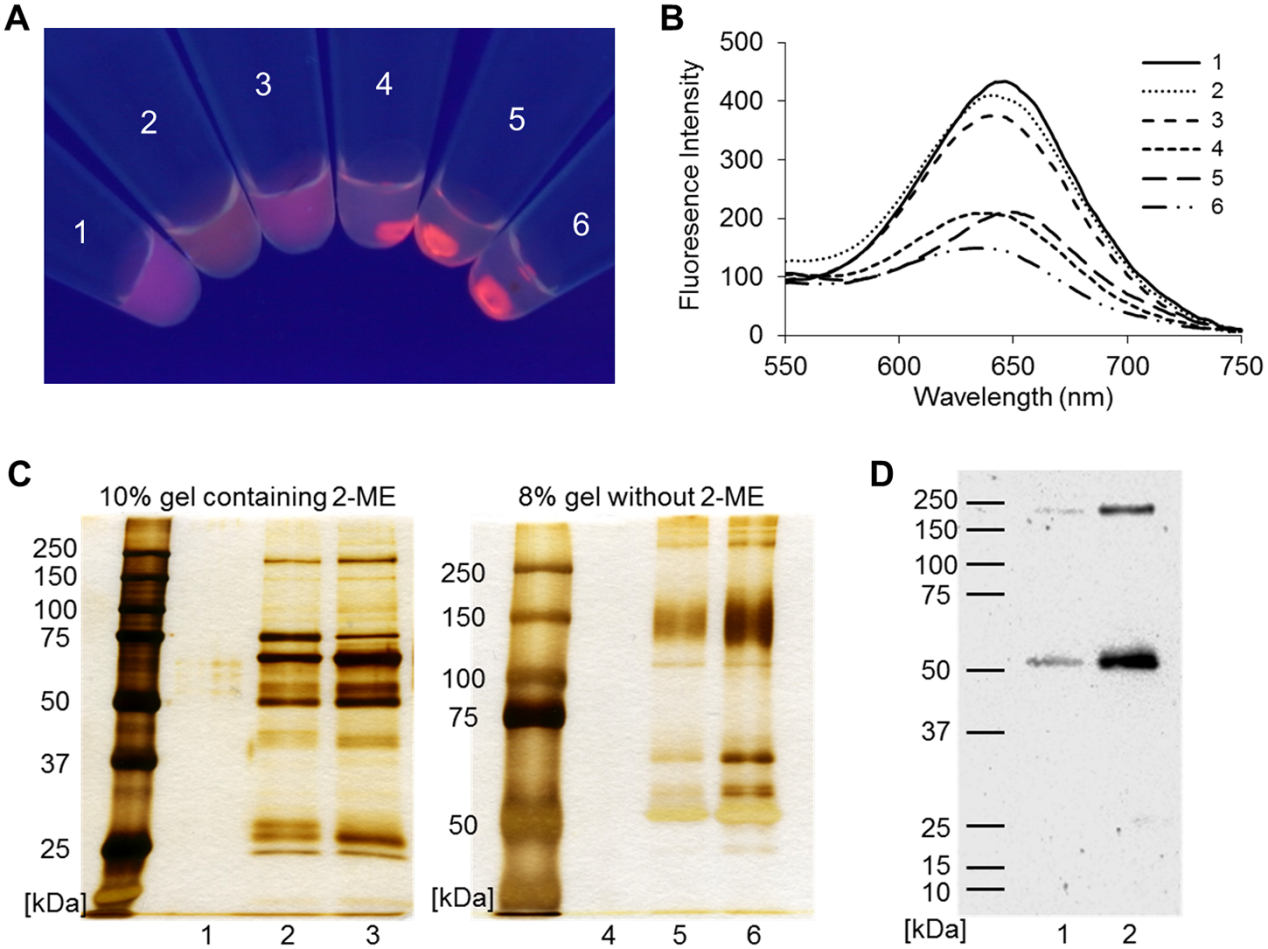
Agglutination assay of GM1-Glc-immobilized fluorescent nanoparticles (GM1-Glc-SFNPs) with sera from GBS patients. (A) Visual image of fluorescent aggregates under UV irradiation (samples 1 and 3: negative for anti-GM1/GD1a antibodies; sample 2: positive for anti-GD1a antibodies; samples 4, 5, and 6: positive for anti-GM1 IgG antibodies). (B) Fluorescence spectra of supernatants monitored by excitation at 360 nm. (C) Silver-stained SDS-PAGE of aggregates obtained from sample 6; 10% gel with 2-mercaptoethanol (2-ME; left) and 8% gel without reducing reagents (right). Lanes 1 and 4: GM1-Glc-SFNP; lanes 2 and 5: aggregates of GM1-Glc-SFNPs; lanes 3 and 6: diluted serum. (D) Western blotting analysis of fluorescent aggregates of GM1-Glc-SFNPs; 10% gel with 2-mercaptoethanol. Lane 1: aggregates of GM1-Glc-SFNPs; lanes 2: diluted serum.

Because SFNPs containing GM1 sugar-chain moieties could agglutinate with a lectin in a structurally specific manner, they were tested on sera from patients with GBS. The sera were supplied from patients that agreed to the clinical or preclinical study performed by the Department of Neurology, Dokkyo Medical University. The study was evaluated and approved by the Ethical Committee of Dokkyo Medical University (No. 1973). Currently, the ELISA method is used to identify anti-GM1 IgG autoantibodies in sera from patients with GBS in Japan [15, 16]. The titers of anti-GM1 antibodies were confirmed and calculated by ELISA. First, the serum samples were simply mixed with GM1-Glc-SFNPs and incubated overnight. Then, they were centrifuged at 14,000 *g* for 5 min. In the case of samples positive for anti-GM1 antibodies, fluorescent aggregates formed after centrifugation (Fig 4A, samples 4–6), and the fluorescence intensity of the supernatant decreased (Fig 4B). In contrast, sera negative for anti-GM1 antibodies did not form any aggregates (Fig 4A, samples 1–3).

**Fig 5:**
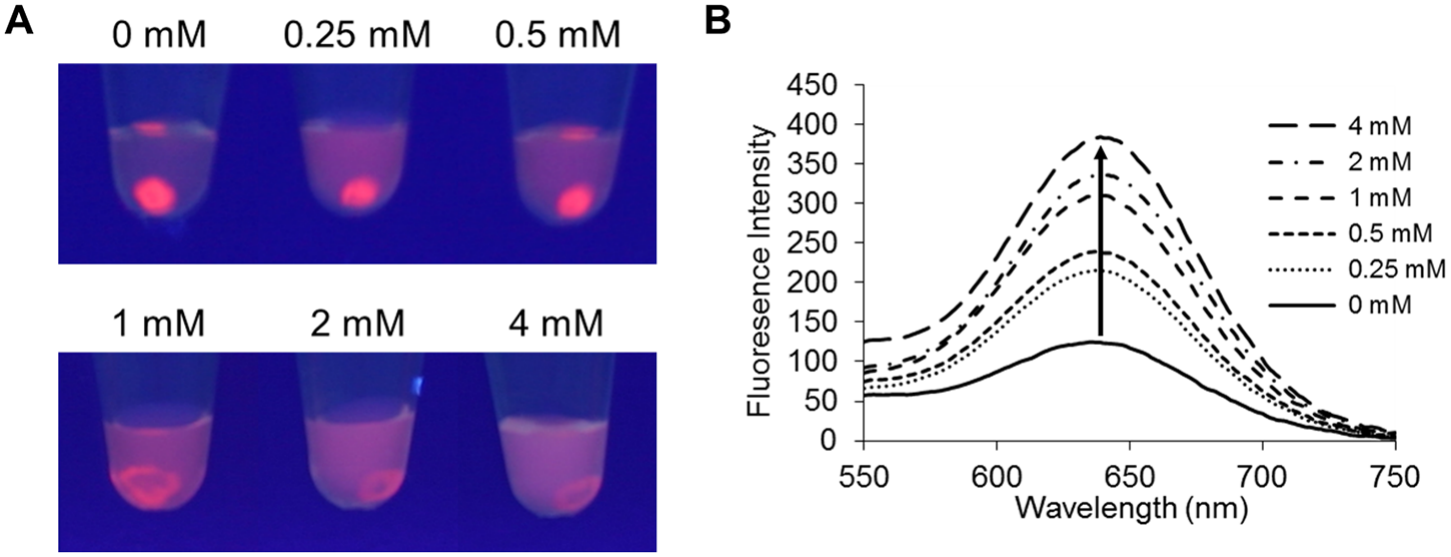
Agglutination inhibition assay of GM1-Glc-immobilized fluorescent nanoparticles (GM1-Glc-SFNPs) with sera from GBS patients. (A) Visual image of the inhibition caused by the addition of free GM1 sugar chains to the agglutination assay (concentration of free GM1 sugar chain: 0 to 4 mM). (B) Fluorescence spectra of the supernatants in the agglutination inhibition assay.

To determine the molecules that were involved in the aggregation, we separated the aggregates and then analyzed them using SDS-PAGE. Four bands at approximately 25, 50, 75, and 150 kDa in a silver-stained gel (Fig 4C) and two bands at approximately 50 and 150 kDa in a western blot with HRP-conjugated goat anti-human IgG (H+L chain specific; Fig 4D) were clearly observed. These bands were interpreted as representing the binding to a fragment of IgG and the binding to a whole IgG, respectively. The addition of free GM1 sugar chains to the agglutination assay inhibited the formation of aggregates in a dose-dependent manner (Fig 5A and 5B). These results indicate that the aggregation occurred via the interaction between SFNPs and anti-GM1 IgG antibodies.

### Optimization of sugar chain densities on SFNPs for agglutination of lectin and autoantibodies from GBS patients

The density of sugar-chain moieties on the QD surfaces is important for the strength of the binding interaction between sugar chains and proteins, and the appropriate sugar chain density can enhance the binding affinity by more than 10- to 1,000-fold [26–32]. Therefore, to obtain the optimal tool for efficient and rapid agglutination assays, density-controlled SFNPs were evaluated as follows: SFNPs with various sugar-chain densities were prepared via a reaction with a mixture of GM1 sugar chain-containing ligand conjugates (**1** or **2**) and a tetraethylene glycol-conjugated monovalent linker molecule (TEG) [20, 33] at ratios of 7:3, 5:5, 3:7, and 1:9.

**Fig 6:**
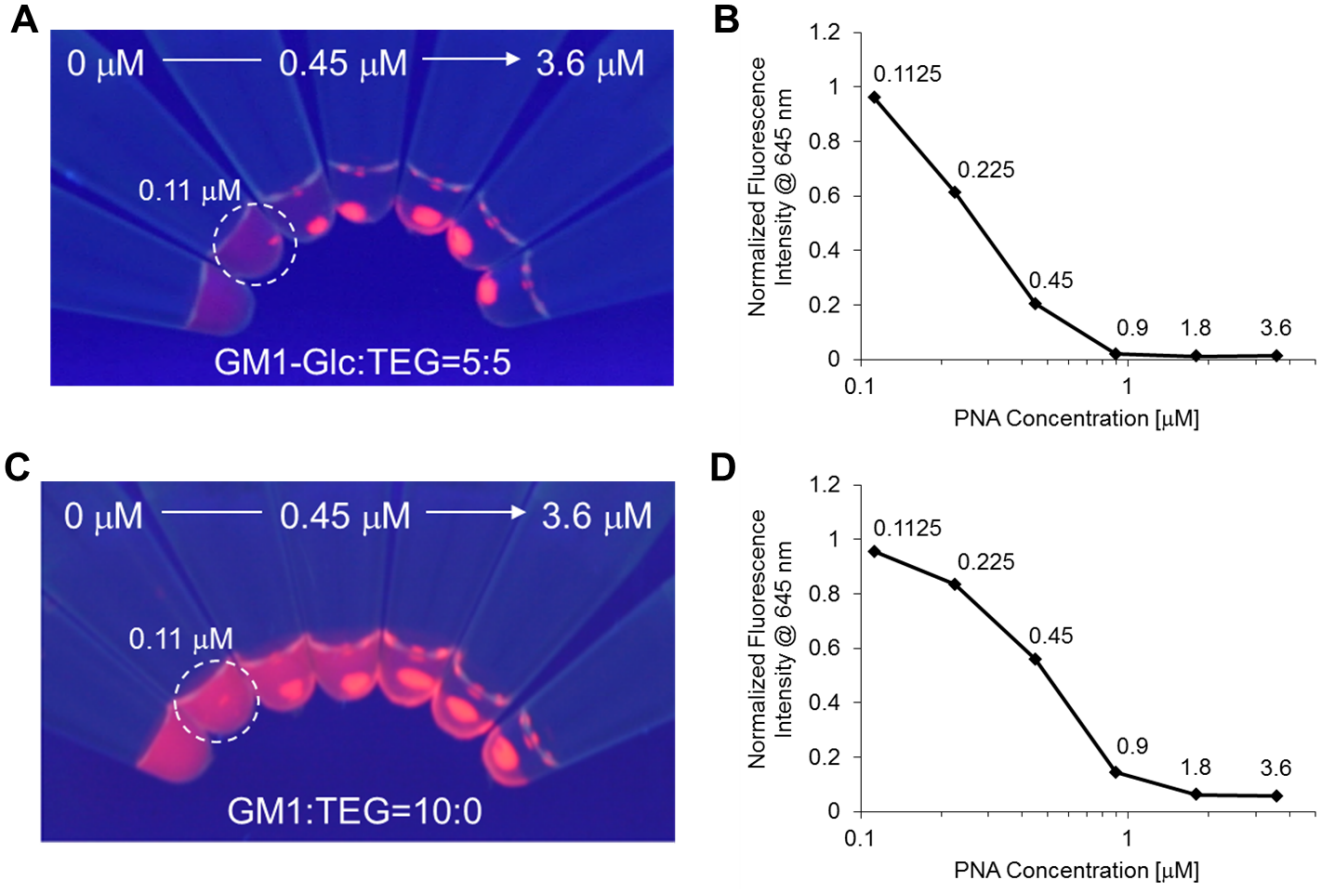
Agglutination assay of PNA using sugar chain-immobilized fluorescent nanoparticles (SFNPs) with different sugar-chain densities. (A) Image from an agglutination assay using GM1-Glc/tetraethylene glycol-conjugated monovalent linker (TEG; 5:5)-SFNPs with peanut agglutinin (PNA). (B) Binding experiment using GM1-Glc/TEG-SFNPs (5:5) to calculate *K*_D_ values using a Scatchard plot. (C) Image from an agglutination assay using GM1-SFNPs with PNA. (D) Binding experiment using GM1-SFNPs to calculate *K*_*D*_ values using a Scatchard plot.

**Fig 7:**
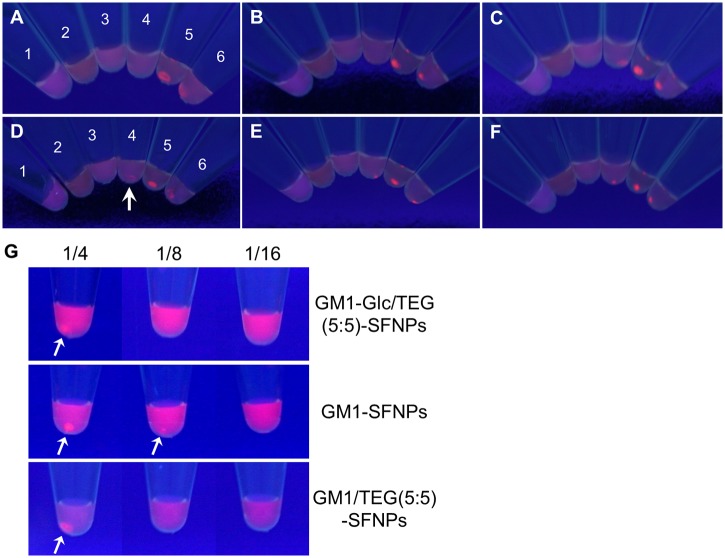
Agglutination assay of GM1-Glc-immobilized fluorescent nanoparticles (GM1-Glc-SFNPs) with different sugar-chain densities and sera from GBS patients. (A–C) Images from an agglutination assay using GM1-Glc-SFNPs and serum from a GBS patient after incubation for (A) 1 h, (B) 3 h, or (C) 12 h. (D–F) Images from an agglutination assay using GM1-Glc/TEG (5:5)-SFNPs and serum from a GBS patient after incubation for (D) 1 h, (E) 3 h, or (F) 12 h. (G) Image from an agglutination assay of GM1-Glc/TEG (5:5)-SFNPs, GM1-SFNPs, or GM1-Glc/TEG-SFNPs using serial dilutions of serum, after incubation for 3 h.

The agglutination assay was performed using PNA at various concentrations (Fig 6A–6D and Table 1). The dissociation constant (*K*_D_) of each SFNP was estimated using a Scatchard plot based on the fluorescence intensity of the supernatants. Aggregate concentrations were visually evaluated to estimate sensitivity. The results show that GM1-Glc/TEG(5:5)-SFNPs and GM1-SFNPs were more effective at aggregation than the other SFNPs. The assay was then performed using sera from patients with GBS (Fig 7A–7F). Interestingly, GM1-Glc/TEG(5:5)-SFNPs were more effective than GM1-Glc-SFNPs in the agglutination assay, producing aggregates after only 1 h of incubation (Fig 7G; see arrow). The assay was also performed using serially diluted serum solutions to compare the detection sensitivities of GM1-Glc/TEG(5:5)-SFNP, GM1-SFNP, and GM1/TEG(5:5)-SFNP. The results show that GM1-SFNP was more effective than GM1-Glc/TEG(5:5)-SFNP or GM1/TEG(5:5)-SFNP after 3 h of incubation.

**Table 1:** Agglutination assay of sugar chain-immobilized fluorescent nanoparticles (SFNPs) with peanut agglutinin.

SFNPs	*K*_*D*_ (nM)	Lowest concentration required to form aggregate (nM)
GM1-Glc-SFNP	298	≤113
GM1-Glc/TEG (7:3)-SFNP	231	≤113
GM1-Glc/TEG (5:5)-SFNP	203	≤113
GM1-Glc/TEG (3:7)-SFNP	1,333	225
GM1-Glc/TEG (1:9)-SFNP	ND	450
GM1-SFNP	980	≤113
GM1/TEG (7:3)-SFNP	2,500	225
GM1/TEG (5:5)-SFNP	ND	225
GM1/TEG (3:7)-SFNP	ND	900
GM1/TEG (1:9)-SFNP	ND	ND

ND, not determined.

### Comparison of agglutination assay with ELISA for testing sera from patients with suspected GBS

The agglutination assay was then evaluated using sera from 100 patients with suspected GBS (S1 and S2 Tables). The ELISA method indicated that 50 of these samples contained anti-GM1 IgG antibodies. In this experiment, GM1-Glc/TEG(5:5)-SFNPs and GM1-SFNPs were used. After 3 h or 12 h of incubation at 4°C in the dark, the mixture of SFNPs and serum was centrifuged at 14,000 *g* for 5 min. Then, the resulting aggregates were visually evaluated under UV irradiation, and the supernatants were analyzed using a fluorescent spectrometer (S2 and S3 Figs). When GM1-Glc/TEG(5:5)-SFNPs were used, 37 of the serum samples formed aggregates after 3 h of incubation, and 2 samples formed aggregates after 12 h of incubation. In contrast, when GM1-SFNPs were used, 43 of the samples produced aggregates after 3 h of incubation (Table 2). Some of the sera that was evaluated as positive for anti-GM1 antibodies using ELISA only formed aggregates with either GM1-Glc/TEG(5:5)-SFNPs or GM1-SFNPs but not with both of them. However, most of the sera that was evaluated as positive for anti-GM1 antibodies using ELISA formed aggregates with GM1-Glc/TEG(5:5)-SFNPs and GM1-SFNPs (S4 Fig). In the case of the sera that was evaluated as negative by ELISA, no aggregates were detected in response to either GM1-Glc/TEG(5:5)-SFNPs or GM1-SFNPs. Overall, the sensitivity of the agglutination assay using SFNPs compared with the ELISA method was as follows: 74% with GM1-Glc/TEG(5:5)-SFNPs after a 3 h incubation, 78% with GM1-Glc/TEG(5:5)-SFNPs after a 12 h incubation, and 86% with GM1-SFNPs after a 3 h incubation, with 100% specificity in all three cases. There is no significant statistical difference between these methods (Table 2). The kappa coefficients for these results against ELISA method were calculated as 0.74, 0.78, and 0.86, respectively.

**Table 2:** Results of the agglutination assay for serum samples using sugar chain-immobilized fluorescent nanoparticles (SFNPs).

	GM1-Glc/TEG(5:5)-SFNPs		GM1-SFNP	
	3 h incubation	12 h incubation	3 h incubation	ELISA
Positive	37	39	43	50
Negative	63	61	57	50
Total	100	100	100	100
*p* value compared with ELISA	*p* = 0.087	*p* = 0.16	*p* = 0.40	–

TEG, tetraethylene glycol-conjugated monovalent linker molecule.

## Discussion

Sugar chain binding antibodies have gained substantial attention as biomarkers due to their crucial roles in various disorders. So far, several detection methods for anti-sugar chain antibodies have been developed including carbohydrate microarray and ELISA [17, 18, 34]. However, those methods are not suitable for point-of-care diagnostics. Thus, we have developed a clinically usable detection method for anti-sugar chain antibodies using our previously developed SFNPs [20]. In this paper, we report a simple and quick point-of-care diagnostic method for GBS, which is as an example of disease involving anti-sugar chain antibodies.

We used the GM1 sugar chain to synthesize two types of sugar chain-ligand conjugates (**1** and **2**) and prepared 10 types of SFNPs possessing different sugar chain structures and densities for detection of anti-GM1 antibody involved in GBS. In the ligand conjugates, a 2,6-diaminopyridine moiety was conjugated with the GM1-Glc or GM1 sugar chain as a linker molecule and was useful for detection during purification because of its fluorescence. The physical properties of all of the prepared SFNPs were similar regarding size, fluorescence, and dispersibility. The presence of the immobilized sugar chains on the SFNPs was qualitatively confirmed using MALDI-TOF/MS analysis and was quantified by the ABEE method. The analysis of lectin binding property indicated that the sugar chains on SFNPs functioned well as ligands for lectin. As in the previous work on lectin-binding interactions, PNA was bound to GM1 more strongly than RCA120 [35].

Because our detection method is based on agglutination between SFNPs and anti-GM1 antibodies in the patients’ sera, one potential problem could be nonspecific binding of the SFNPs with other serum proteins. However, as described above, SFNPs bind to lectins in a specific manner and did not form aggregates with BSA, indicating the binding specificity of the immobilized sugar chain. Previous studies using ELISA to detect anti-GM1 antibodies showed that anti-GM1 IgG antibodies specifically bind to GM1 and are detectable, even at low concentrations [15, 16]. Thus, we also evaluated the agglutination assay using sera from patients with GBS, and could visually detect aggregates of SFNPs and anti-GM1 antibodies under UV irradiation after several hours of incubation.

Next, to decrease the diagnostic time and increase the sensitivity of the assay, we optimized the sugar chain density and structure on the SFNPs. Gangliosides generally exist in a cluster form in so-called lipid rafts on cell membranes where they regulate various biofunctions [36, 37]. Appropriate distances between sugar chains are required for the clustering effect, which increases the interactions between sugar chains and proteins [38–40]. In addition, the sizes of the nanoparticles and IgG antibodies are also an important factor in an agglutination assay. The sizes of the CdTe/CdS nanoparticles, which were used as the core components, and the sizes of the SFNPs are approximately 5 nm and 9 nm in diameter, respectively. The size of an IgG antibody is approximately 15 nm in diameter, and the binding sites in the antibody are approximately 10 nm away from each other [41]. Thus, it has been suggested that inter-nanoparticle interactions of antibodies are preferred over the intra-nanoparticle interactions. In the agglutination assay using the patient’s sera, GM1-SFNPs were more effective than GM1-Glc/TEG(5:5)-SFNPs in most cases, although GM1-Glc/TEG(5:5)-SFNPs were preferable in a few cases. These results indicate that there were optimal sugar chain structure and density for effective interaction with antibodies. The numbers of sugar chains on GM1-SFNPs and GM1-Glc-SFNPs were estimated as 165 and 135 per particle, respectively. We are currently using computer simulations to perform a detail analysis of the interactions between various densities of immobilized sugar structures and IgG antibodies; this analysis will be reported in a separate manuscript. Further fine-tuning of the sugar structure and density on the SFNPs may be required; however, the results described in this study suggest that more effective diagnostic methods may be available with a combination of various SFNPs immobilized with different sugar chain structures and densities.

SFNPs can be used for the quantitative assay. However, the agglutination assay in the current study using sera from GBS patients was qualitative. Because purified anti-GM1 IgG antibody which forms aggregates with SFNPs was not available, the amount of the antibody was not able to determine. In addition, there was no correlation between the titers of IgG to gangliosides and the grade of severity of GBS (S1 Table). Thus, it was difficult to assess the disease from amount of aggregates. In the present study, when GM1-SFNPs were used, the kappa coefficient for our system compared with the ELISA method was 0.86, which indicated good agreement in the detection of anti-GM1 antibodies. Compared with ELISA, our diagnostic method using SFNPs was simple because it only required incubation of the SFNPs with patient serum, and it was fast (within 3 h). Thus, our system can be used in clinical facilities (point-of-care) to diagnose GBS.

As the simple diagnostic methods for GBS, latex beads agglutination assay has been reported by Alaedini, *et al* [42]. However, this method needs an optical microscope to observe aggregates. In contrast, our method can visually diagnose under UV light without any special equipment. Additionally, our method can be used for detection of wide variety of sugar chain binding antibodies because the sugar chain structure on the SFNPs can be easily modified to various types of sugar chain. A number of anti-sugar chain antibodies have been found in sera from patients with diseases, such as cancer, rheumatoid arthritis and others [19]. SFNPs technology can be applied to the point-of-care diagnostics of various types of diseases involving anti-sugar-chain antibodies. Our method, therefore, would be a versatile platform for the point-of-care diagnostics of various diseases.

## Conclusions

We demonstrated a rapid and simple diagnostic test to detect anti-GM1 antibodies in sera from patients with suspected GBS. Our method of detecting anti-GM1 antibodies is simpler and faster than the ELISA method and may be utilized onsite to confirm GBS. Because our SFNPs can be easily prepared with various sugar chains, disease-tailored SFNPs would be powerful point-of-care diagnostic tools.

## Supporting Information

S1 Fig Characterization of GM1-sugar immobilized fluorescent nanoparticles (GM1-SFNPs). (A) MALDI-TOF MS analysis of GM1-SFNPs. The detected peak was *m/z*: 1442.73 [M-H]^−^, corresponding to GM1-f-mono. (B) Interaction analysis between GM1-SFNPs and lectins. (left) Visual image of the mixture of GM1-SFNPs and proteins under UV irradiation. (right) Fluorescence spectrum of the supernatant monitored by excitation wavelength at 360 nm.
(TIF)

S2 Fig Agglutination assay of GM1-Glc/TEG(5:5)-SFNPs with 100 serum samples. (A) The image of agglutination assay with GM1-Glc/TEG(5:5)-SFNPs and anti-GM1 IgG antibody positive sera (top) and anti-GM1 IgG antibody negative sera (bottom) after 3 h incubation. (B) Fluorescent intensity of supernatant monitored by excitation wavelength at 360nm.
(TIF)

S3 Fig Agglutination assay of GM1-SFNPs with 100 serum samples. (A) The image of agglutination assay with GM1-SFNPs and anti-GM1 IgG antibody positive sera (top) and anti-GM1 IgG antibody negative sera (bottom) after 3 h incubation. (B) Fluorescent intensity of supernatant monitored by excitation wavelength at 360nm.
(TIF)

S4 Fig Agglutinating ability against either GM1-Glc/TEG(5:5)-SFNPs or GM1-SFNPs after 12 h incubation. (TIF)

S1 Table Hughes functional grading scale and titers of IgG to various gangliosides including GM1. (DOC)

S2 Table Titers of IgG to various gangliosides in sera of patients used for negative control. (DOC)
