# Supplementary figures and images for: Visual Detection of Human Antibodies Using Sugar Chain-Immobilized Fluorescent Nanoparticles: Application as a Point of Care Diagnostic Tool for Guillain-Barré Syndrome
Source: PLoS One. 2015 Sep 17;10(9):e0137966. doi: 10.1371/journal.pone.0137966 (PMC4574945; doi:10.1371/journal.pone.0137966)

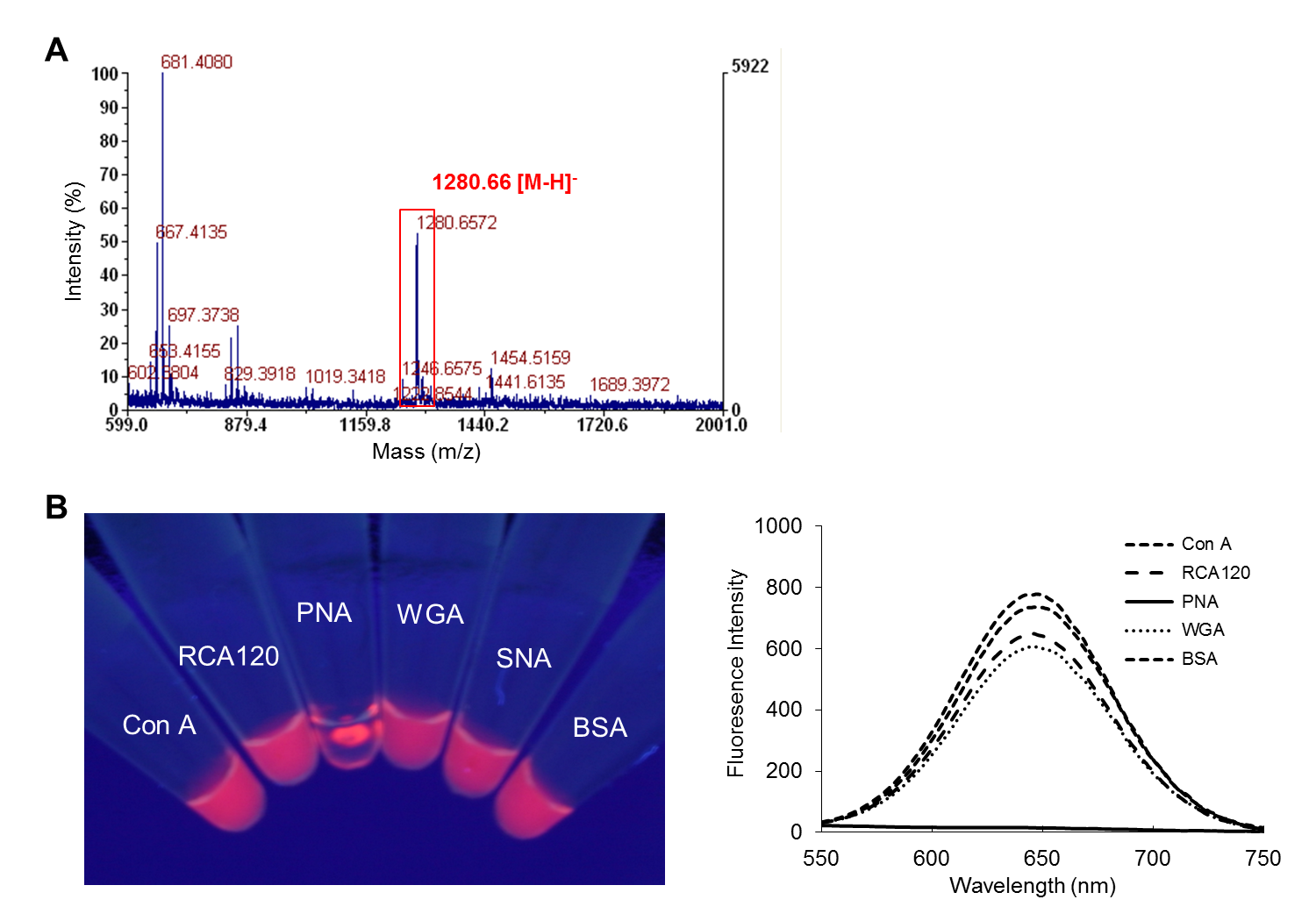

Supplement: S1 Fig — (A) MALDI-TOF MS analysis of GM1-SFNPs. The detected peak was m/z: 1442.73 [M-H]−, corresponding to GM1-f-mono. (B) Interaction analysis between GM1-SFNPs and lectins. (left) Visual image of the mixture of GM1-SFNPs and proteins under UV irradiation. (right) Fluorescence spectrum of the supernatant monitored by excitation wavelength at 360 nm. (TIF) [file pone.0137966.s001.tif]

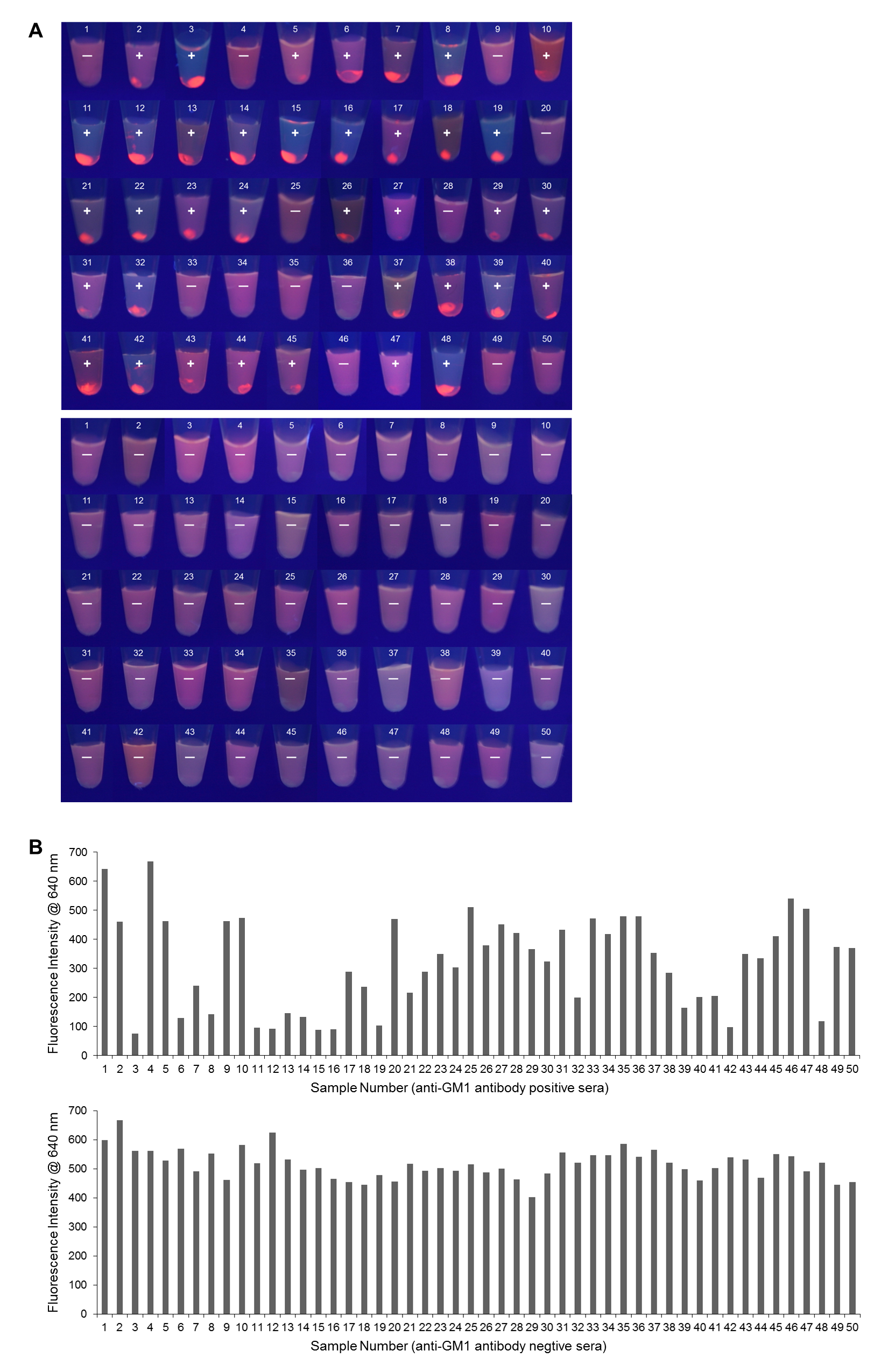

Supplement: S2 Fig — (A) The image of agglutination assay with GM1-Glc/TEG(5:5)-SFNPs and anti-GM1 IgG antibody positive sera (top) and anti-GM1 IgG antibody negative sera (bottom) after 3 h incubation. (B) Fluorescent intensity of supernatant monitored by excitation wavelength at 360nm. (TIF) [file pone.0137966.s002.tif]

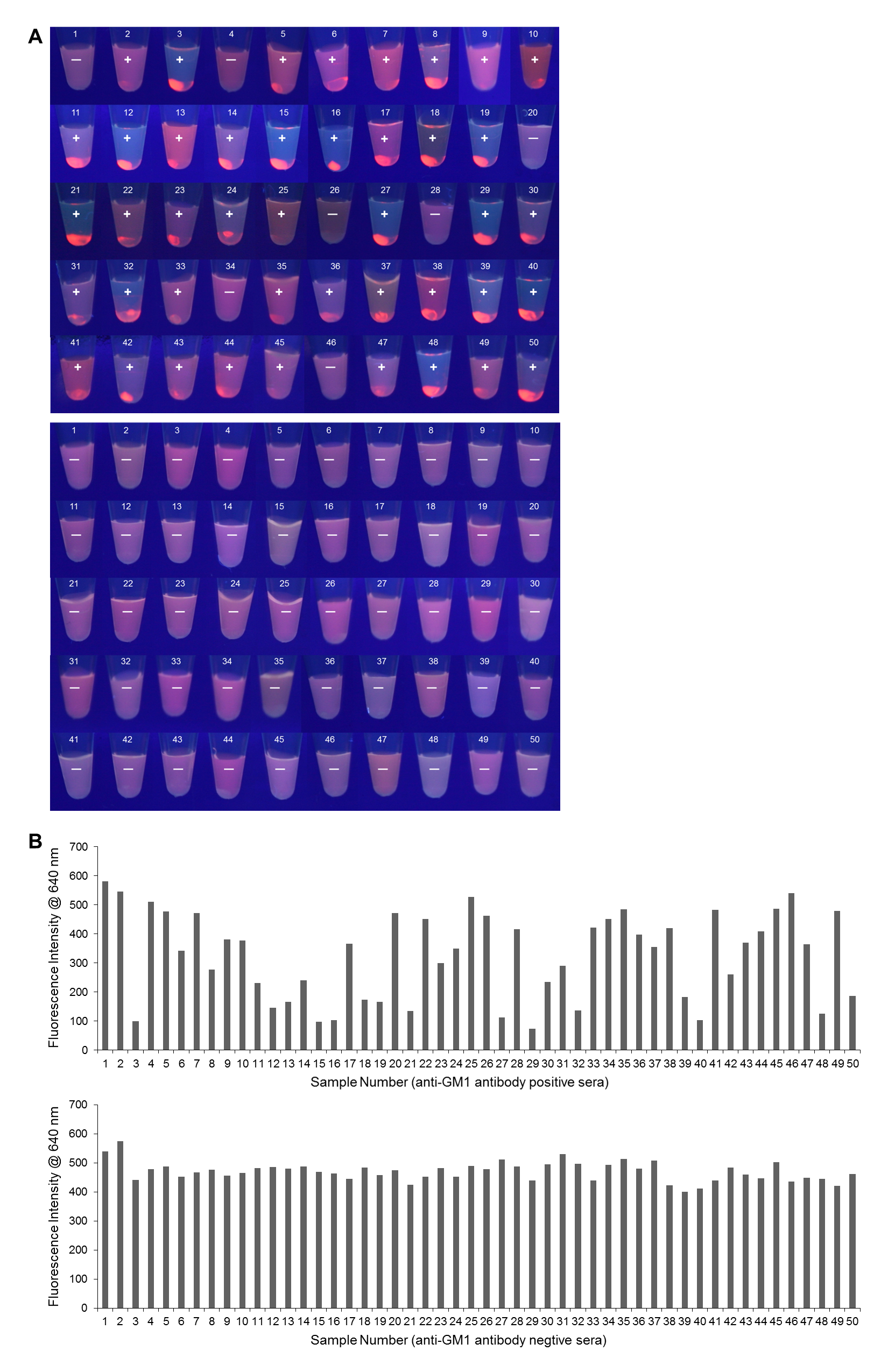

Supplement: S3 Fig — (A) The image of agglutination assay with GM1-SFNPs and anti-GM1 IgG antibody positive sera (top) and anti-GM1 IgG antibody negative sera (bottom) after 3 h incubation. (B) Fluorescent intensity of supernatant monitored by excitation wavelength at 360nm. (TIF) [file pone.0137966.s003.tif]

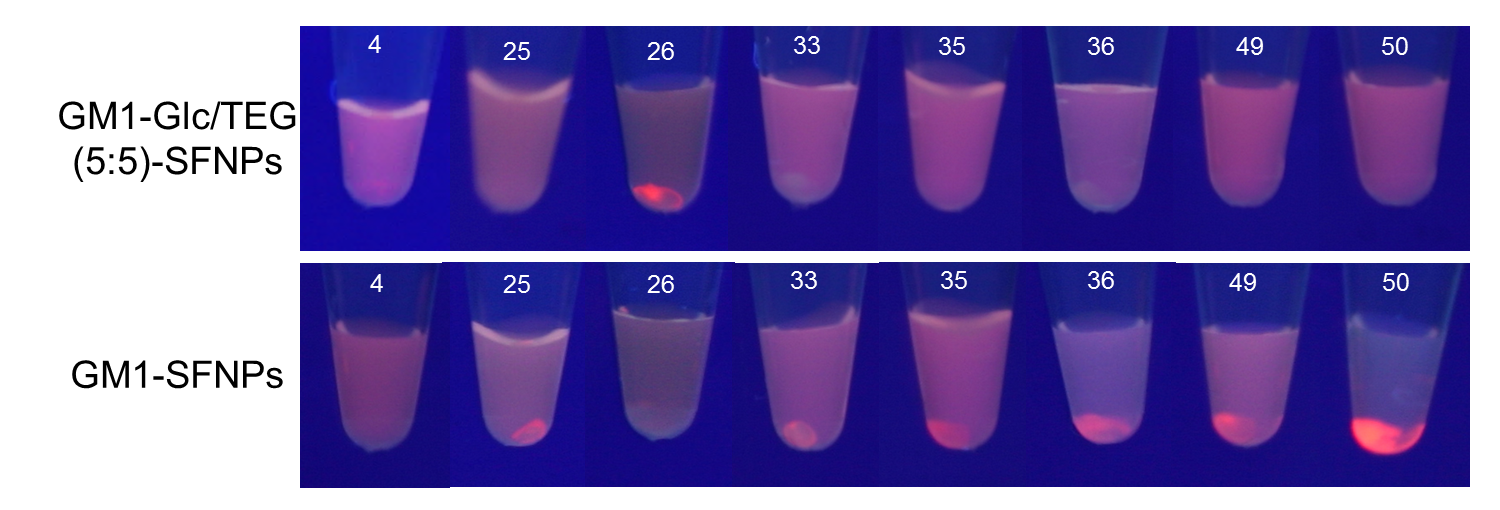

Supplement: S4 Fig — (TIF) [file pone.0137966.s004.tif]
