# Supplementary material for: Visual Detection of Human Antibodies Using Sugar Chain-Immobilized Fluorescent Nanoparticles: Application as a Point of Care Diagnostic Tool for Guillain-Barré Syndrome
Source: PLoS One. 2015 Sep 17;10(9):e0137966. doi: 10.1371/journal.pone.0137966 (PMC4574945; doi:10.1371/journal.pone.0137966)
Supplement: S1 Table — (DOC) [file pone.0137966.s005.doc]

**S1 Table. Hughes functional grading scale and titers of IgG to various gangliosides including GM1.**

| **Sample Number** | **Hughes functional grading scale*^a^*** | **Titers to gangliosides*^b^*** | | | | | | |
| --- | --- | --- | --- | --- | --- | --- | --- | --- |
|  |  | **GM1** | **GM1b** | **GD1a** | **GalNAc-GD1a** | **GD1b** | **GT1a** | **GQ1b** |
| **1** | - | 6+ | – | 6+ | – | 6+ | 1+ | 1+ |
| **2** | 3 | 6+ | 2+ | 5+ | 2+ | 6+ | – | 1+ |
| **3** | 2 | 6+ | 1+ | 2+ | 1+ | 1+ | 1+ | 1+ |
| **4** | 4 | 6+ | 2+ | 6+ | 5+ | 6+ | 1+ | 1+ |
| **5** | 2 | 6+ | – | 4+ | – | 4+ | – | – |
| **6** | 4 | 6+ | 1+ | 6+ | 2+ | 6+ | 1+ | 1+ |
| **7** | 3 | 4+ | – | 1+ | 1+ | 1+ | – | – |
| **8** | 2 | 6+ | – | 2+ | 5+ | – | – | – |
| **9** | 2 | 6+ | – | 3+ | 1+ | 3+ | – | – |
| **10** | 4 | 6+ | – | 1+ | 1+ | 1+ | 6+ | 1+ |
| **11** | 4 | 5+ | – | 1+ | – | 2+ | – | – |
| **12** | 4 | 6+ | 1+ | 6+ | 4+ | 6+ | 1+ | 1+ |
| **13** | 4 | 6+ | 3+ | 6+ | 1+ | 6+ | 6+ | 6+ |
| **14** | 2 | 6+ | – | 1+ | – | 1+ | – | – |
| **15** | 4 | 6+ | – | 3+ | – | 1+ | 1+ | – |
| **16** | 4 | 6+ | – | 6+ | 2+ | 6+ | 5+ | – |
| **17** | 2 | 3+ | – | – | – | – | 4+ | 1+ |
| **18** | 4 | 6+ | – | – | – | 1+ | – | – |
| **19** | 3 | 6+ | – | – | – | 3+ | 1+ | – |
| **20** | 4 | 4+ | – | 5+ | – | 6+ | 2+ | 1+ |
| **21** | 4 | 3+ | 1+ | – | – | 4+ | – | – |
| **22** | 4 | 6+ | – | – | 2+ | 4+ | 1+ | – |
| **23** | 3 | 6+ | – | – | – | 6+ | – | – |
| **24** | 2 | 6+ | 4+ | 1+ | 1+ | 6+ | – | – |
| **25** | 4 | 6+ | – | 4+ | 4+ | 5+ | – | – |
| **26** | 4 | 5+ | – | – | – | – | – | – |
| **27** | 4 | 3+ | – | 1+ | 1+ | 5+ | – | – |
| **28** | 4 | 5+ | 1+ | 4+ | 1+ | 3+ | – | – |
| **29** | 3 | 6+ | – | 3+ | – | – | – | – |
| **30** | 3 | 6+ | – | – | – | 1+ | 1+ | – |
| **31** | 4 | 6+ | 1+ | 1+ | 1+ | 4+ | 1+ | 1+ |
| **32** | 4 | 6+ | – | – | 1+ | 1+ | – | – |
| **33** | 4 | 6+ | – | – | – | 1+ | – | – |
| **34** | 4 | 3+ | – | – | – | 1+ | – | – |
| **35** | 2 | 6+ | – | 3+ | – | 5+ | 1+ | – |
| **36** | 4 | 6+ | – | 1+ | 1+ | 6+ | – | – |
| **37** | 4 | 5+ | – | – | 4+ | 5+ | – | – |
| **38** | 4 | 3+ | – | – | – | 1+ | – | – |
| **39** | 4 | 6+ | – | 1+ | 3+ | 6+ | 1+ | – |
| **40** | 4 | 5+ | – | 4+ | 1+ | 6+ | – | – |
| **41** | 3 | 3+ | – | – | 1+ | 1+ | 1+ | – |
| **42** | 2 | 5+ | – | 3+ | 6+ | 1+ | – | – |
| **43** | 3 | 5+ | – | 1+ | – | 6+ | – | – |
| **44** | 2 | 6+ | – | 4+ | – | 4+ | 4+ | – |
| **45** | 3 | 3+ | 1+ | – | – | – | – | – |
| **46** | 2 | 4+ | 3+ | – | – | 1+ | – | – |
| **47** | 4 | 6+ | – | 2+ | 3+ | – | – | – |
| **48** | 4 | 6+ | – | 2+ | 2+ | – | 1+ | – |
| **49** | 4 | 3+ | – | – | – | – | 6+ | – |
| **50** | 4 | 4+ | – | 2+ | – | 5+ | 1+ | – |

*^a^* Hughes functional grading scale: Grade 0, normal; Grade 1, minimal signs and symptoms, able to run; Grade 2, ambulates independently; Grade 3, able to walk 5 m with aid; Grade 4, bed bound; Grade 5, requires assisted respiration; Grade 6 dead.

*^b^* Titers to gangliosides were determined by ELISA. Each patient serum was diluted at 1:500, and titers were graded as described previously (Kimoto K, *et al*., Neurology 2006; 67: 1837-1843): An optical density at 492 nm of less than 0.1 was judged to be negative. The optical density of 0.1 to 0.5 was categorized as 1+; 0.5 to 1.0, 2+; 1.0 to 1.5, 3+; 1.5 to 2.0, 4+; 2.0 to 2.5, 5+; and 2.5 or more, 6+.
