# Supplementary material for: Visual Detection of Human Antibodies Using Sugar Chain-Immobilized Fluorescent Nanoparticles: Application as a Point of Care Diagnostic Tool for Guillain-Barré Syndrome
Source: PLoS One. 2015 Sep 17;10(9):e0137966. doi: 10.1371/journal.pone.0137966 (PMC4574945; doi:10.1371/journal.pone.0137966)
Supplement: S2 Table — (DOC) [file pone.0137966.s006.doc]

**S2 Table. Titers of IgG to various gangliosides in sera of patients used for negative control.**

| **Sample Number** | **Titers to gangliosides*^a^*** | | | | | | |
| --- | --- | --- | --- | --- | --- | --- | --- |
|  | **GM1** | **GM1b** | **GD1a** | **GalNAc-GD1a** | **GD1b** | **GT1a** | **GQ1b** |
| **1** | – | – | – | – | – | – | – |
| **2** | – | – | – | – | – | – | – |
| **3** | – | – | – | – | – | – | – |
| **4** | – | – | – | – | – | – | – |
| **5** | – | – | – | – | – | – | – |
| **6** | – | – | – | – | – | 1+ | – |
| **7** | – | – | – | – | – | – | – |
| **8** | – | – | – | – | – | – | – |
| **9** | – | – | – | – | – | – | – |
| **10** | – | – | – | – | – | – | – |
| **11** | – | – | – | – | – | – | – |
| **12** | – | – | – | – | – | – | – |
| **13** | – | – | – | – | – | – | – |
| **14** | – | – | – | – | – | – | – |
| **15** | – | – | – | – | – | – | – |
| **16** | – | – | – | – | – | – | – |
| **17** | – | N.D.*^b^* | – | N.D.*^b^* | – | N.D.*^b^* | – |
| **18** | – | N.D.*^b^* | – | N.D.*^b^* | – | N.D.*^b^* | – |
| **19** | – | – | – | – | – | – | – |
| **20** | – | – | – | – | – | – | – |
| **21** | – | – | – | – | – | – | – |
| **22** | – | – | – | – | – | – | – |
| **23** | – | N.D.*^b^* | – | N.D.*^b^* | – | N.D.*^b^* | – |
| **24** | – | N.D.*^b^* | – | N.D.*^b^* | – | N.D.*^b^* | – |
| **25** | – | N.D.*^b^* | – | N.D.*^b^* | – | N.D.*^b^* | – |
| **26** | – | N.D.*^b^* | – | N.D.*^b^* | – | N.D.*^b^* | – |
| **27** | – | N.D.*^b^* | – | N.D.*^b^* | – | N.D.*^b^* | – |
| **28** | – | N.D.*^b^* | – | N.D.*^b^* | – | N.D.*^b^* | – |
| **29** | – | N.D.*^b^* | – | N.D.*^b^* | – | N.D.*^b^* | – |
| **30** | – | N.D.*^b^* | – | N.D.*^b^* | – | N.D.*^b^* | – |
| **31** | – | – | – | – | 1+ | – | – |
| **32** | – | – | – | – | – | – | – |
| **33** | – | – | – | – | – | – | – |
| **34** | – | – | – | – | – | – | – |
| **35** | – | – | – | – | – | 1+ | – |
| **36** | – | N.D.*^b^* | N.D.*^b^* | N.D.*^b^* | – | N.D.*^b^* | – |
| **37** | – | N.D.*^b^* | N.D.*^b^* | N.D.*^b^* | 1+ | N.D.*^b^* | 1+ |
| **38** | – | N.D.*^b^* | N.D.*^b^* | N.D.*^b^* | 1+ | N.D.*^b^* | 1+ |
| **39** | – | N.D.*^b^* | N.D.*^b^* | N.D.*^b^* | – | N.D.*^b^* | – |
| **40** | – | N.D.*^b^* | N.D.*^b^* | N.D.*^b^* | – | N.D.*^b^* | – |
| **41** | – | – | – | 1+ | 1+ | – | 1+ |
| **42** | – | – | – | – | – | – | – |
| **43** | – | – | – | – | – | – | – |
| **44** | – | – | – | – | – | 1+ | – |
| **45** | – | – | – | – | – | – | – |
| **46** | – | N.D.*^b^* | N.D.*^b^* | N.D.*^b^* | 1+ | N.D.*^b^* | – |
| **47** | – | N.D.*^b^* | N.D.*^b^* | N.D.*^b^* | – | N.D.*^b^* | – |
| **48** | – | N.D.*^b^* | N.D.*^b^* | N.D.*^b^* | – | N.D.*^b^* | – |
| **49** | – | N.D.*^b^* | N.D.*^b^* | N.D.*^b^* | – | N.D.*^b^* | – |
| **50** | – | N.D.*^b^* | N.D.*^b^* | N.D.*^b^* | – | N.D.*^b^* | – |

*^a^* Titers to gangliosides were determined by ELISA. Each patient serum was diluted at 1:500, and titers were graded as described previously (Kimoto K, *et al*., Neurology 2006; 67: 1837-1843): An optical density at 492 nm of less than 0.1 was judged to be negative. The optical density of 0.1 to 0.5 was categorized as 1+; 0.5 to 1.0, 2+; 1.0 to 1.5, 3+; 1.5 to 2.0, 4+; 2.0 to 2.5, 5+; and 2.5 or more, 6+.

*^b^* N.D. : not determined.
